# A process-tracing protocol for evaluating ethnic identity mobilization in subnational dynamics of national elections^☆^

**DOI:** 10.1016/j.mex.2026.104203

**Published:** 2026-10-13

**Authors:** Cut Maya Aprita Sari, Ibnu Phonna Nurdin, Annisah Putri

**Affiliations:** a Department of Political Science, Faculty of Social Sciences and Political Sciences, Universitas Syiah Kuala, Banda Aceh, Indonesia; b Department of Sociology, Faculty of Social Sciences and Political Sciences, Universitas Syiah Kuala, Banda Aceh, Indonesia

**Keywords:** Process tracing, Causal mechanisms, Explaining outcomes, Subnational campaign dynamics, Presidential elections, Ethnic identity politics

## Abstract

This methodology article presents adjustments to previously established process tracing methods, resulting in a set of technical protocols for the "explaining outcome" variant, specifically tailored to unpack the causal mechanisms underlying ethnic identity mobilization within the subnational dynamics of national elections. While the influence of identity politics is widely recognized, its transformation from campaign narrative to electoral victory is often lacking in empirical validation and procedural transparency. The protocols in explaining outcome-process tracing are characterized by:

• A causal mechanism that captures how candidate strategies interact with subnational gatekeeper entities and activities to drive electoral victory.

• The Hoop Test and Smoking Gun Test are applied to evaluate diagnostic evidence supporting the research propositions.

• The technique generates a visual timeline that distinguishes between technocratic actions and identity-based mobilization actions to demonstrate the causal mechanism.

This protocol was validated through a longitudinal case study spanning five Indonesian presidential election cycles (2004–2024) in Aceh, ensuring that qualitative political analysis remains auditable and replicable to a degree comparable with quantitative designs for studying identity politics globally.

## Graphical abstract

**Figure:**
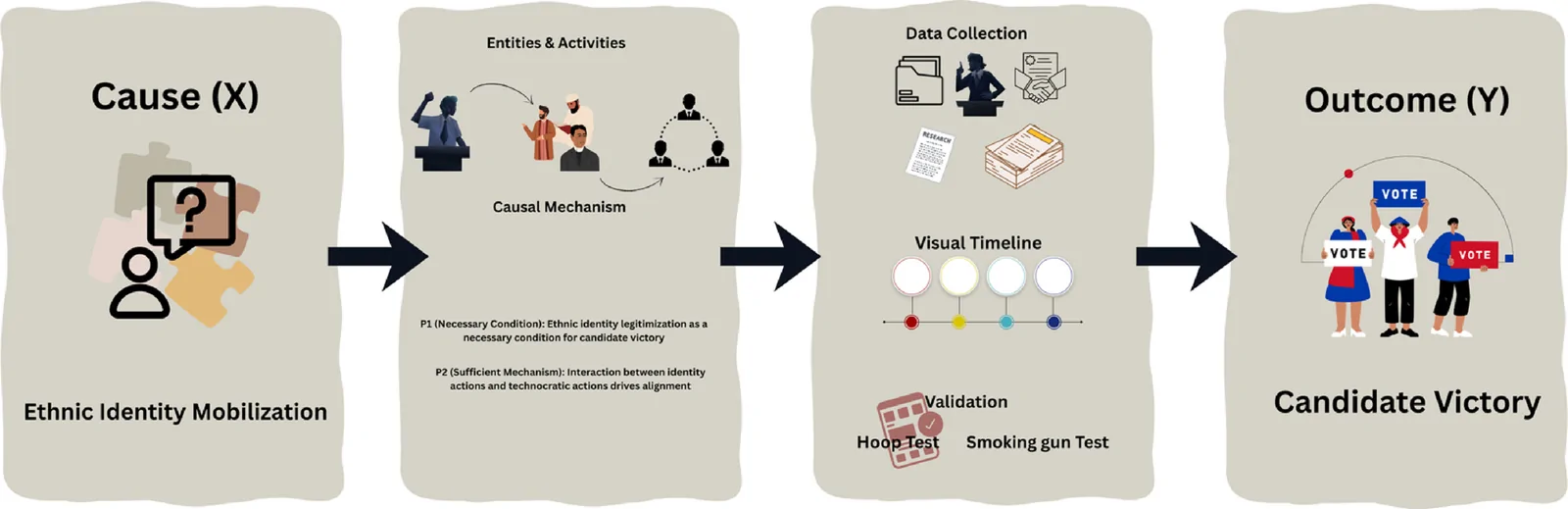

**Specifications table**

**Table:** 

**Subject area**	Social Sciences (Political Science/Sociology)
**More specific subject area**	Political Science, Ethnic Studies, Electoral Studies, Qualitative Methods
**Name of your method**	Process-Tracing Protocol for Explaining-Outcome Variant in Subnational Dynamics of National Elections
**Name and reference of original method**	Process-Tracing in Social Sciences Beach, D., & Pedersen, R. B. (2013). Process- Tracing Methods: Foundations and Guidelines. The University of Michigan Press. Bennett, A., & Checkel, J. T. (2014). Process Tracing: From Metaphor to Analytic Tool.
**Resource availability**	Supplementary dataset S2

## Background

In political science and comparative sociology, understanding how ethnic identity politics influences province-level political alignment in subnational dynamics of national elections remains crucial [[1], [2], [3]]. However, empirical studies often treat ethnic identity as a static, descriptive attribute or a simple independent variable, thus failing to explain how identity-based campaign narratives actually translate into electoral victories [4,5]. Conventional quantitative models, on the other hand, often fail to capture informal elite bargaining, spatial ethnic geography, and symbolic transactions in autonomous or post-conflict regions [6,5]. Conversely, unstructured qualitative studies risk becoming purely descriptive historical narratives that lack systematic causal inference [7,8].

To bridge the gap between theoretical propositions and qualitative empirical rigor, this article aims to provide a step-by-step protocol for the explaining-outcome variant of process tracing in subnational dynamics of national elections [[9], [10], [11]]. Process tracing serves as a relevant within-case method for identifying dynamic causal mechanisms linking initial conditions to outcomes [4,12]. Adapted from existing methodological handbooks [9,10], this protocol provides a structured workflow that transforms qualitative data such as speeches, political contracts, media archives, and cultural rituals into verifiable diagnostic evidence.

The protocol addresses several ongoing methodological issues in studies of subnational dynamics of national presidential election cycles by operationalizing this strategy. First, it integrates qualitative process-tracing diagnostic tests, namely Hoop Tests and Smoking Gun Tests [13,14], to analyze diagnostic evidence, thereby overcoming the frequent lack of explicit operational criteria for evidence evaluation [11,12]. Second, it eliminates temporal uncertainty in situations where normal narrative process tracing cannot clearly identify causal sequences. Drawing on visual mapping techniques used in historical process tracing [15], the protocol introduces a dual-box visual timeline to determine whether identity mobilization directly preceded elite endorsement cascades and political alignment rather than technocratic promises. Finally, by standardizing the identification of local gatekeepers (such as regional parties, religious leaders, and traditional elites), the protocol offers a replicable and adaptable analytical framework for researchers studying ethnic identity or autonomy-related contexts globally [1,6].

## Method details

Process tracing is a qualitative research method used to identify the causal mechanism linking a cause (X) to a specific outcome (Y) [9]. Without attempting to build universal theoretical models, this protocol operationalizes the case-centric explaining-outcome variant to craft a contextually grounded, minimally sufficient causal explanation for subnational campaign dynamics. In subnational electoral research, causal mechanisms function as transmission pathways linking national candidate strategies, local broker networks, and political alignment. To transform this analysis into a transparent and replicable framework, this methodology translates explaining-outcome process tracing into a structured seven-step protocol.

### What is customized here

While standard process-tracing handbooks provide general principles for evaluating causal mechanisms, they offer limited operational guidance for subnational campaign environments during national elections [9,10]. This protocol complements existing operational guidance by introducing a seven-step protocol tailored to subnational identity mobilization. Table 1 outlines the methodological customization of the proposed protocols.

**Table 1:** Methodological customizations in the proposed seven-step protocol.

**Protocol Step**	**Standard Process-Tracing Guidance** [9,10].	**Protocol Customization/Addition**	**Methodological Problem Addressed**
Step 1: Scope & Outcome	Defines outcome (Y) as a generic historical case without multi-level specifications.	Restricts scope to subnational campaign dynamics and province-level alignment within national elections.	Reduces risk of unit-of-analysis mismatch with local elections.
Step 2: Hypothesizing	Conceptualizes mechanisms as abstract theoretical chains of entities and activities.	Establishes a dual-channel mechanism distinguishing Technocratic vs. Identity-Based mobilization.	Disentangles competing campaign drivers in ethnically sensitive settings.
Step 3: Data Collection	Recommends iterative data gathering until qualitative confidence or saturation is reached.	Integrates five batch stopping rule, digitization bias reporting, and permanent web preservation services.	Addresses vague saturation claims, mitigates digitization bias in older cycles, and prevents link rot.
Step 4: Evidence Coding	Categorizes empirical evidence into generic typologies (pattern, sequence, trace, and account evidence).	Implements three evidence typologies (Rhetorical, Formal, Ritualistic) with explicit verification requirements (Primary vs. Secondary Formal).	Reduces risk of over-crediting unverified media reports of elite pacts and captures local ritual dynamics.
Step 5: Sequence Mapping	Maps empirical evidence using narrative temporal chronologies or linear flowcharts.	Applies dual-box visual timeline mapping (Shaded grey = Technocratic; White = Identity).	Clarifies temporal ordering between campaign events and endorsement cascades to reduce ambiguity.
Step 6: Diagnostic Testing	Applies Bayesian diagnostic tests (Straw in the Wind, Hoop, Smoking Gun, Doubly Decisive).	Calibrates diagnostic tests to subnational elite gatekeeping and mandates counterfactual testing on losing cases.	Establishes systematic criteria for evaluating necessary (P1) and sufficient (P2) conditions.
Step 7: Causal Inference	Synthesizes evidence narratively to draw case-centric causal explanations.	Provides a standardized inferential framework linking endorsement cascades and identity legitimacy to outcome Y.	Offers a structured, reproducible template for case-centric explaining-outcome conclusions.

### The process-tracing protocol for explaining outcomes in subnational dynamics of national elections

The explaining-outcome process-tracing approach for studying subnational dynamics of national elections is driven by the specific objective of explaining complex election outcomes across diverse regional contexts [9]. In province-level campaign dynamics, particularly in post-conflict or autonomous regions, substantial technocratic incentives (e.g., infrastructure budgets or special autonomy funds) often coexist with the mobilization of entrenched ethnic identities [16]. It takes an eclectic approach that combines systematic causal mechanisms with local institutional dynamics to explain why identity-based narratives often eclipse technocratic programs.

In research on subnational dynamics of national elections, causal mechanisms serve as complex transmission pathways linking national candidate strategies, local broker networks, and political alignment [9]. To transform this case-centered analysis into a transparent, rigorous, and replicable scientific framework, this methodology translates the logic of explaining-outcome process tracing into a structured seven-step protocol:

*Step 1: Conceptualizing the causal chain*

The protocol begins by conceptualizing the causal chain and formulating research questions (RQs) that direct inquiry toward intermediary mechanisms. In applying this protocol to subnational dynamics of national presidential elections, researchers can specify three core research questions: (RQ1) How does the mobilization of ethnic identity by political candidates influence electoral outcomes in the target subnational region? (RQ2) What specific causal mechanisms drive the impact of ethnic identity politics on candidate success at the local level? (RQ3) How do identity mobilization variables interact with non-identity factors (e.g., economic promises and candidate resources) to determine subnational political alignment?

When conceptualizing “ethnic identity,” researchers must first consider the specific socio-cultural and historical attributes of the subnational region under study. For instance, when this framework is applied to Aceh, ethnic identity emerges as a combination of the historical memory of conflict and the Islamic faith; both, therefore, serve as elements in understanding Aceh’s subnational political alignment.

*Step 2: Formulating working propositions*

Following conceptualization, hypothesized causal mechanisms are translated into testable working propositions. The propositions are structured using Beach & Pedersen’s [9] entity-activity formalism, where each part of a mechanism is explicitly specified as a concrete actor (entity) engaging in a causal action (activity). Proposition 1 (P1) posits ethnic identity legitimization as a baseline necessary condition: national political candidates (Entity) must obtain symbolic identity legitimization from subnational identity gatekeepers (Activity) to establish baseline candidate viability in the target subnational region. Failure to secure this baseline legitimization eliminates a candidate's viability (as evaluated via Hoop Tests).

Proposition 2 (P2) posits the entity-activity mechanism as a sufficient causal chain: candidate victory (Y) is driven by an integrated three-part entity-activity sequence that combines ethnic identity-based legitimization with formal alignment and technocratic commitments:

Part 1 sacred & symbolic legitimization): national candidates (entity 1) engage in sacred or traditional legitimization practices alongside subnational identity gatekeepers (entity 2, activity 1). (for instance, in our application to Aceh, this manifests as candidate participation in *peusijuk* ceremonies and royal tomb pilgrimages with regional *ulamas*).
Part 2 (institutional & broker alignment): subnational party elites and local brokers (entity 3) issue formal joint endorsement declarations and sign political pacts (activity 2).
Part 3 (technocratic policy framing): national candidates and campaign teams (entity 1) articulate targeted subnational policy guarantees, such as fiscal autonomy, decentralization pledges, or regional development commitments (activity 3). (In the Aceh context, this translates into promises of special autonomy fund extensions.) To ensure replicability, Table 2 presents the full entity-activity decomposition for P1 and P2.

**Table 2:** Entity-activity mechanism specification and expected diagnostic evidence.

**Proposition / Mechanism Part**	**Entity (Actor)**	**Activity (Causal Action)**	**Evidence Typology & Characteristics**	**Expected Diagnostic Evidence (Operational Indicators)**	**Diagnostic Test Type**	**Timeline Box Placement**
P1: Baseline Identity Legitimization (Necessary Condition)	National Candidates & Subnational Identity Gatekeepers	Seeking and conferring regional symbolic/identity blessing for candidate viability.	Ritualistic Evidence (Traditional rituals, sacred site visits, public blessings)	Media coverage and visual archives documenting candidate participation in regional traditional or religious legitimization rituals.	Hoop test (Failure eliminates candidate viability)	White Box
P2- Part 1: Sacred & Symbolic Legitimization	National Candidates & Regional Cultural/Religious Gatekeepers	Performing legitimization ceremonies and conferring public symbolic approval.	Ritualistic Evidence (Symbolic endorsement & sacred site veneration)	Official statements of endorsement, joint cultural/religious gatherings, and blessing ceremonies by respected regional gatekeepers.	Smoking Gun Test (Confirms identity-based legitimization)	White Box
P2 - Part 2: Institutional & Broker Alignment	Subnational Party Leadership & Local Elite Brokers	Formally declaring political coalition pacts and activating regional support networks.	Formal Evidence (Signed coalition MoUs, formal endorsement declarations)	Cross-verified media reports on signed political pacts, joint press conferences, and official local party endorsements.	Smoking Gun Test (Confirms formal broker alignment)	White Box
P2 - Part 3: Technocratic Policy Framing	National Candidates & National Campaign Teams	Articulating subnational fiscal, autonomy, or development policy promises.	Rhetorical Evidence (Speech framing, campaign pledges, policy discourse)	Transcripts of campaign speeches, media interview archives, and public commitments regarding regional fiscal autonomy or infrastructure programs.	Hoop test (Verifies presence of policy framing)	Grey Box

*Step 3: Collecting secondary data*

Data collection relies on secondary sources such as historical archives, media records, campaign materials, memoranda of understanding (MoUs), and policy documents to trace whether hypothesized causal steps are reflected in the actual empirical sequence of events [7]. Researchers must unpack the "black box" linking candidate strategies to election outcomes by breaking it down into observable intermediate steps.

To ensure longitudinal integrity and eliminate selection bias across national presidential election cycles, data collection adheres to a concrete operational rule for information saturation: data gathering from academic journals, archival reports, speech transcripts, signed agreements, and news outlets must continue until a batch of five consecutive additional sources yields no new coded evidence items, candidate behaviors, symbol uses, or previously unidentified local actors.

Furthermore, researchers analyzing multi-decade periods must account for digitization bias, for instance, by reporting the total number of sources and media details for each election cycle. Additionally, to prevent link rot and ensure the reproducibility of long-term protocols, researchers should record access dates and archive all online news URLs using permanent web preservation services (such as the Internet Archive or Perma.cc).

*Step 4: Identifying diagnostic evidence*

Once data is gathered, researchers systematically identify diagnostic evidence to test the validity of working propositions [9,12]. Diagnostic evidence must provide sufficient detail to verify whether specific entities (such as candidates or local brokers) engaged in specific causal activities (such as identity framing or political pacts).

Diagnostic evidence is categorized into three typologies that serve as an operational codebook for classifying campaign evidence: (1) Rhetorical Evidence, which can be transcripts of campaign speeches, campaign audio/video, official slogans, and recorded social media statements. (2) Formal Evidence, including Documented Memorandum of Understanding (MoUs), signed political pacts, written policy pledges, and formal endorsement letters. Researchers must apply a strict verification requirement to formal evidence: agreements obtained in full primary text carry maximal diagnostic weight for Smoking Gun Tests. If the MoU or pact is known only second-hand through media reporting, it must be coded as Secondary-Reported Formal Evidence and assigned lower diagnostic weight, as media summaries may omit or distort specific contractual nuances. (3) Ritualistic Evidence, such as news coverage and visual documentation of a candidate's physical participation in local cultural ceremonies, wearing traditional attire, or visiting sacred historical sites.

Rhetorical and ritualistic evidence are primary diagnostic inputs for testing P1 through Hoop Tests. Passing a Hoop Test is a necessary condition for validating P1; if a presidential candidate relies solely on national developmentalist rhetoric and fails to deploy ethnic identity, it fails the Hoop Test for P1, thereby eliminating identity mobilization as a causal driver for that specific candidate's campaign.

When conducting research in polarized environments, formal evidence (e.g., signed MoUs with local elites) provides stronger diagnostic weight for P2 than general media reporting, as it directly demonstrates a deliberate transactional link in the causal mechanism. Where feasible, researchers are strongly encouraged to supplement secondary archives with elite interviews (e.g., with local party campaign directors, party secretariats, or local gatekeepers). Primary interviews serve as a crucial triangulation layer to verify informal bargaining dynamics and solidify Smoking Gun claims for P2.

To prevent selection bias, researchers must apply the evidence identification and coding instrument identically to both winning and losing candidates. Comparative coding and reporting of losing cases provides the essential counterfactual variation required to execute valid diagnostic tests.

*Step 5: Developing a visual timeline*

The fifth step entails organizing diagnostic evidence into a chronological timeline covering the campaign period. A campaign event is defined as a specific public activity on a single date. Events reported by only one news source require cross-verification against official itineraries or secondary archives before inclusion. The primary objective is to map the temporal sequence and ensure that the cause (X) precedes the outcome (Y). Standard process tracing applications often rely solely on lengthy narrative text, which can obscure chronological patterns and comparative intensity. To address this limitation, this protocol adapts a visual mapping approach developed by Sari et al. [15]. While Sari et al. [15] mapping historical conflict across four broad phases (1500–2005) on a narrative timeline, this protocol modifies it by: (1) adding entity-activity coding, (2) introducing a dual-box scheme (shaded grey for technocratic vs. white for identity actions), and (3) shifting the temporal scale to daily campaign events.

Researchers can construct a dual-box visual timeline mapped along a shared chronological axis. Box A (shaded grey box-technocratic actions): Infrastructure development pledges, economic policy announcements, administrative campaign events, and policy debate performances. Box B (white box-ethnic identity actions): Visits to sacred or historical sites, religious or cultural endorsements, speeches emphasizing symbolic identity, and the conferral of traditional titles. For campaign events involving both technocratic actions and ethnic identity actions in a single day, the protocol disaggregates the event into distinct coding units: technocratic actions are coded in Box A, whereas ethnic identity actions are coded in Box B. This disaggregation ensures that the dual-box scheme maintains strictly mutually exclusive categories without requiring an ambiguous hybrid category.

An exemplar of this dual-box visual timeline is presented in Fig. 1, which shows the use of ethnic identity during the 2024 presidential election campaign in Aceh as a technical demonstration. Each significant campaign event is mapped chronologically alongside key electoral milestones, candidate/coalition details, and references to empirical sources. By visually distinguishing technocratic actions from identity mobilization actions, researchers can systematically verify whether Box B events cluster before elite endorsement cascades and final electoral outcomes relative to Box A events.

**Fig. 1:**
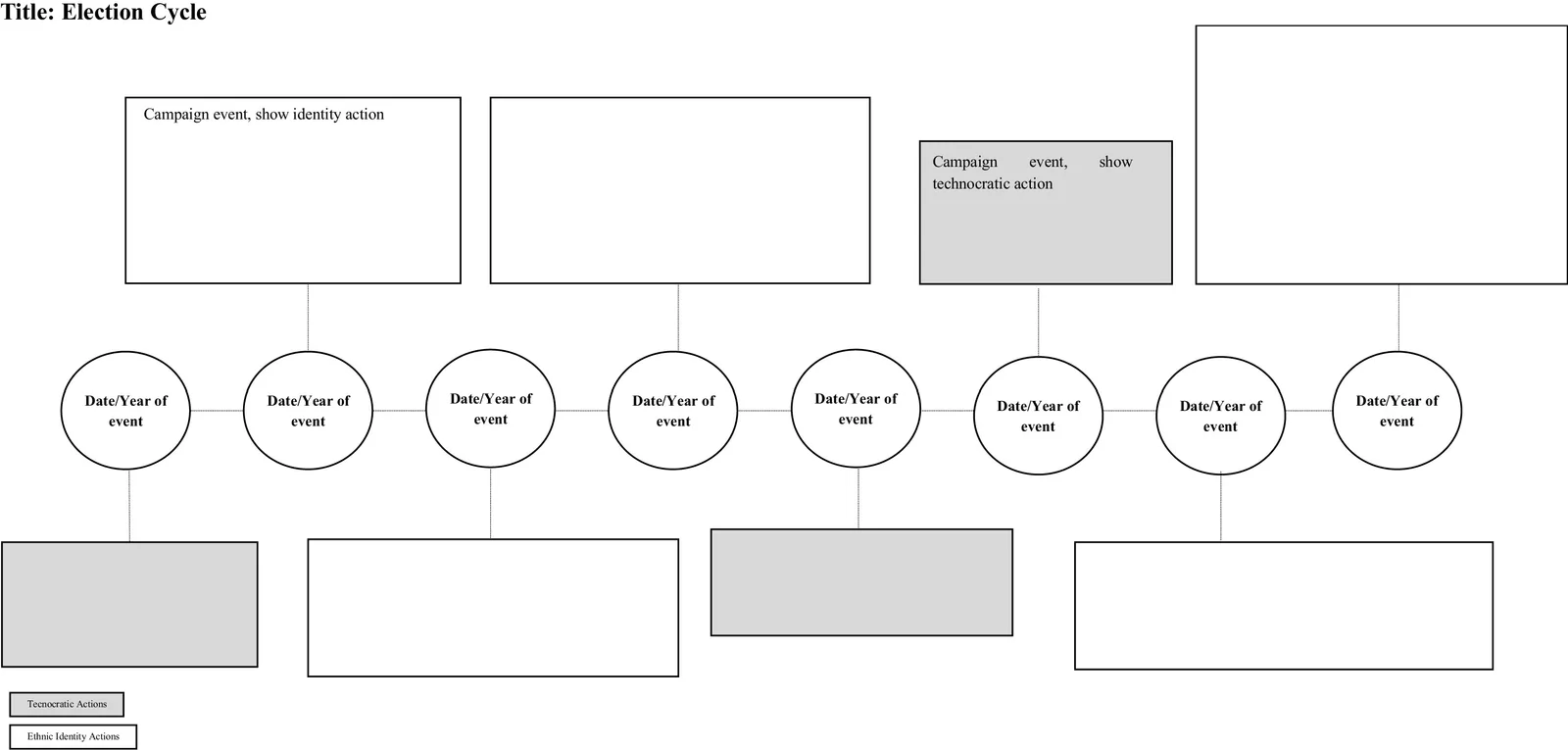
Election Cycle.

To address reverse causality and endogeneity (such as elite support reflecting a bandwagoning phenomenon), the protocol incorporates three explicit procedures:

(A) Linking campaign events to independently dated polls, where available. If continuous polling data is unavailable, researchers should limit their claims to observable temporal aspects. Particularly by verifying whether mobilization events cluster before endorsement cascades.
(B) Including at least one observation that deviates from the general pattern. Such as an elite endorsing a candidate who ultimately loses, or an endorsement not followed by a political alignment.
(C) Specifying the temporal patterns that would falsify the proposed mechanism. This applies if waves of support consistently precede identity-related actions, indicating elites were responding to the electoral environment rather than actively shaping it.

To evaluate protocol reliability, researchers should independently code a random subsample of campaign events, typically 10–20% of the total event dataset [17]. In our empirical validation, based on 70 secondary data sources, three independent coders (e.g., members of the research team) evaluated a representative 20% subsample of campaign events (n = 14) across election cycles. This test yielded high inter-coder agreement (Cohen's κ = 0.86), categorized as almost perfect agreement, with initial discrepancies resolved through consensus [18,19].

*Step 6: Assessing the evidence*

Coded diagnostic evidence is evaluated using qualitative process-tracing diagnostic tests [13,14]. Specifically, the Hoop Test and the Smoking Gun Test are used to determine whether working propositions are confirmed or eliminated [11]. The Hoop Test is used to evaluate P1 and to assess whether candidate identity framing is a necessary condition for electoral victory [11]. To pass the Hoop Test, empirical evidence must show that winning candidates consistently adopted ethnic identity markers. If data reveals that a candidate who completely ignored ethnic identity markers secured an electoral majority, P1 fails the Hoop Test and is eliminated from the causal chain.

A Hoop Test is only useful if the tested condition varies among candidates; if an action or feature is present across all candidates regardless of whether they win or lose, it carries no diagnostic value and should not be used as a Hoop Test. Furthermore, the Smoking Gun Test is used to evaluate P2 and assesses whether the four-part entity-activity chain linking national candidates, regional parties, and local gatekeepers is empirically instantiated [13,14]. Documented formal MoUs, political bargaining agreements, or public legitimization rituals serve as smoking-gun evidence. Passing the Smoking Gun Test provides strong confirmatory support that the interaction within the entity-activity chain forms a valid causal mechanism driving the electoral outcome.

Researchers need to consider the functional differences between these two tests. The Hoop Test serves to eliminate a proposition; failure causes the proposition to be discarded, whereas passing merely allows it to survive. Conversely, the "smoking-gun test" serves to confirm a proposition; passing it provides strong support, but failure does not automatically invalidate the proposition, as the crucial piece of evidence might not be recorded in historical archives.

*Step 7: Performing analytical explanations*

The final stage integrates the visual timeline and validated logic test results into a coherent analytical narrative. The narrative synthesis proceeds backward from the final election result to verify whether validated empirical evidence forms a continuous, logically sound causal chain. For replication, researchers must carefully identify the specific actors (entities) and their legitimizing actions (activities) that mediate identity sentiment into political alignment and electoral outcomes (as specified in Table 2).

To demonstrate the complete operational execution of the seven-step protocol, Table 3 presents a worked end-to-end example chain using one campaign event from the 2024 election cycle in Aceh.

**Table 3:** Worked end-to-end operational example chain.

**Operational Stage**	**Execution & Analytical Output**
1. Raw Secondary Source Excerpts (Chronological Campaign Sequence)	1 November 2023: "Anies visited North Aceh to participate in joint shalawat and dhikr, state Aceh’s identity as a 'capital region', and make a pilgrimage to Sultan Malikussaleh Tomb. Simultaneously, three local parties (PAS, PDA, SIRA) declared joint support, while Anies made policy commitments regarding Special Autonomy Fund (*Otsus*) extension." (Supplementary dataset S2).
2. Coded Evidence Items (Chronological Sequential Indexing)	• Joint shalawat and royal tomb pilgrimage. • Joint party endorsement declaration by PAS, PDA, and SIRA. • Campaign discourse on "capital region" identity and *otsus* policy promises.
3. Evidence Typology Assignment	• Ritualistic: Sacred religious participation and tomb veneration. • Formal: Public coalition endorsement by subnational political parties. • Rhetorical: Campaign speech framing and fiscal policy promises.
4. Dual-Box Timeline Placement	• White Box (Identity actions): Religious legitimization driving local party alignment. • Grey Box (Technocratic actions): *Otsus* fiscal autonomy policy commitment.
5. Diagnostic Test Application	Smoking Gun Test for P2: Evaluates whether simultaneous ritual legitimization provided a sufficient condition to secure the multi-party endorsement cascade.
6. Causal Inference Output	Passes Test: Confirms that local party consolidation on 1 November 2023 was directly facilitated by dual-channel mobilization, combining ethno-religious legitimization with technocratic fiscal guarantees.

## Method validation

The proposed protocol was empirically validated through a longitudinal case study of subnational campaign dynamics in Aceh across five Indonesian presidential election cycles (2004–2024). The validation workflow systematically progressed from gathering 70 secondary data sources to diagnostic coding based on explicit rules, dual-box visual timeline mapping, and qualitative diagnostic testing to establish a validated causal mechanism.

Data collection reliability was validated through information saturation and systematic triangulation across 70 secondary data sources spanning 2004 to 2024, including campaign speech transcripts, news archives, formal political contracts, and candidate itineraries, as shown in Supplementary Dataset S2 [20]. Raw event data were systematically cross-referenced and validated using established political science literature on Aceh’s post-conflict settlement, special autonomy, and *ulama* authority [16,21]. To explicitly address digitization bias over the two-decade window, sources were cataloged by election cycle: 2004 (n = 12), 2009 (n = 14), 2014 (n = 15), 2019 (n = 16), and 2024 (n = 13). While earlier cycles relied more heavily on scanned print archives, official election reports, and early online media, contemporary cycles drew from dense digital streams. Information saturation was verified by applying the stopping rule: data collection for each cycle ceased when five consecutive additional sources were collected.

Shifts in local voter support were tracked using qualitative indicators from regional and national press reports, specifically monitoring public rally attendance figures, the timing of endorsements by local *ulama*, and contemporary media reporting on candidate momentum following identity-mobilization events.

The diagnostic strength of empirical evidence regarding both winning and losing presidential candidates is evaluated using qualitative process-tracing diagnostic tests [13,14]. The Hoop Test evaluated P1 regarding brokered identity framing. Across five election cycles, winning candidates (such as SBY-JK in 2004, SBY-Boediono in 2009, Prabowo-Hatta in 2014, Prabowo-Sandi in 2019, and Anies-Muhaimin in 2024) secured identity-based legitimacy mediated by local gatekeepers and won in the provinces. Conversely, losing candidates who lacked local elite intermediaries (such as Megawati-Hasyim in 2004, Megawati-Prabowo in 2009, and Jokowi-Ma'ruf in 2019) suffered significant vote deficits in Aceh.

Consequently, P1 survived the Hoop Test, failing to be disconfirmed as a necessary condition. Furthermore, the Smoking Gun Test evaluated P2 by revealing documented political MoUs, signed pacts, and public endorsements between national candidates and local identity brokers, including *ulamas* and regional political parties such as Partai Aceh. Because the primary text of these documents was not publicly available, they were coded as secondary formal evidence, based on cross-verified news reports, with their diagnostic weight adjusted accordingly. The formal agreements and ritualistic legitimizations provided strong confirmatory support for P2, indicating that local gatekeeper entities act as key brokers connecting national candidates to province-level political alignment through legitimizing activities.

Finally, temporal sequence verification through dual-box visual timelines created for each election cycle (Fig. 2A-E and supplementary table S1) confirmed that cause (X) strictly preceded outcome (Y). By explicitly color-coding campaign events into distinct visual boxes, the timeline clearly distinguished between Technocratic Actions (shaded grey boxes, such as infrastructure promises and Special Autonomy Fund discussions) and Identity-Based Mobilization Actions (white boxes, such as *peusijuk*/ blessing rituals, sacred site visits, and cleric endorsements). The validation revealed that clusters of white-box events consistently preceded elite endorsement cascades and political alignment relative to shaded grey-box events. Together, these empirical findings demonstrate the protocol's utility and inferential rigor in providing a systematic, reproducible explanation of subnational campaign dynamics in national presidential elections.

**Fig. 2:**
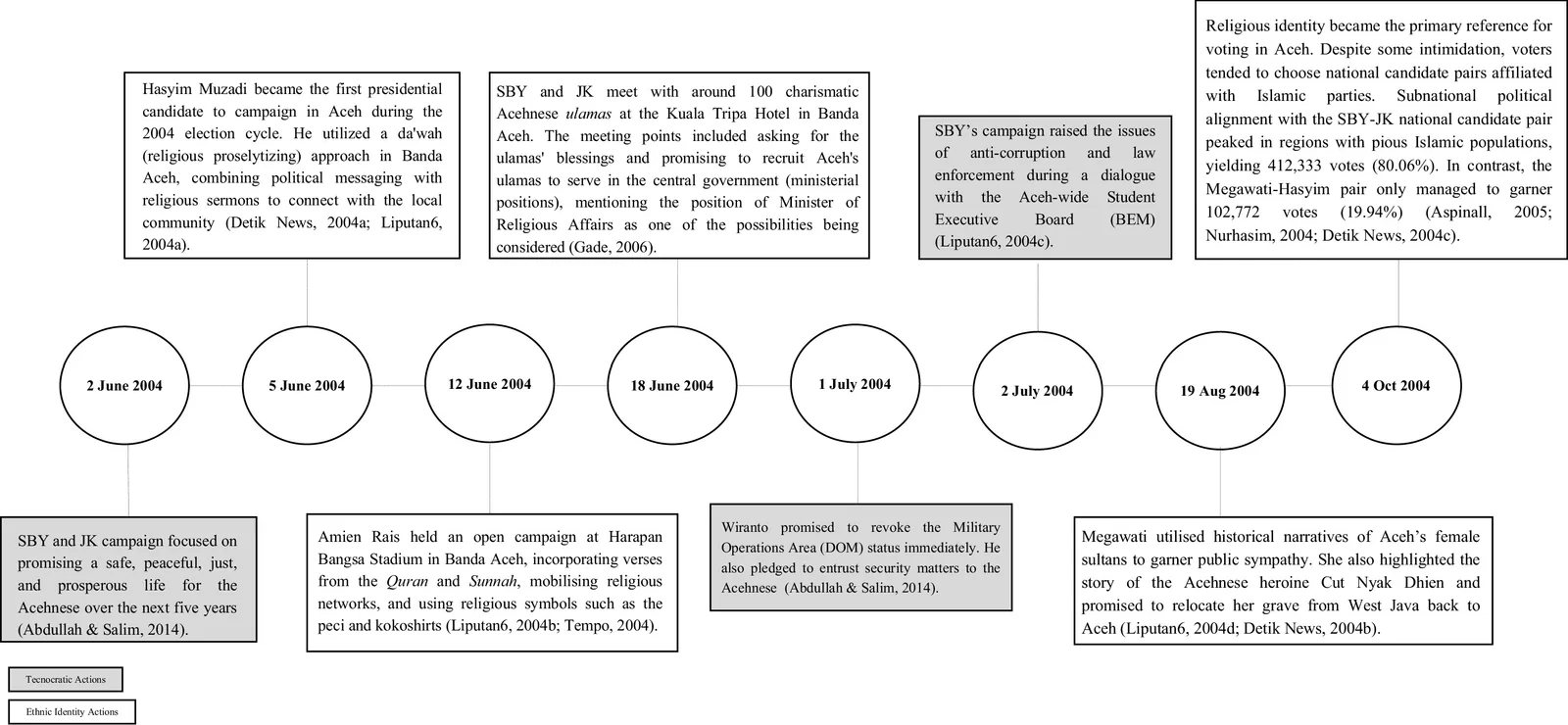

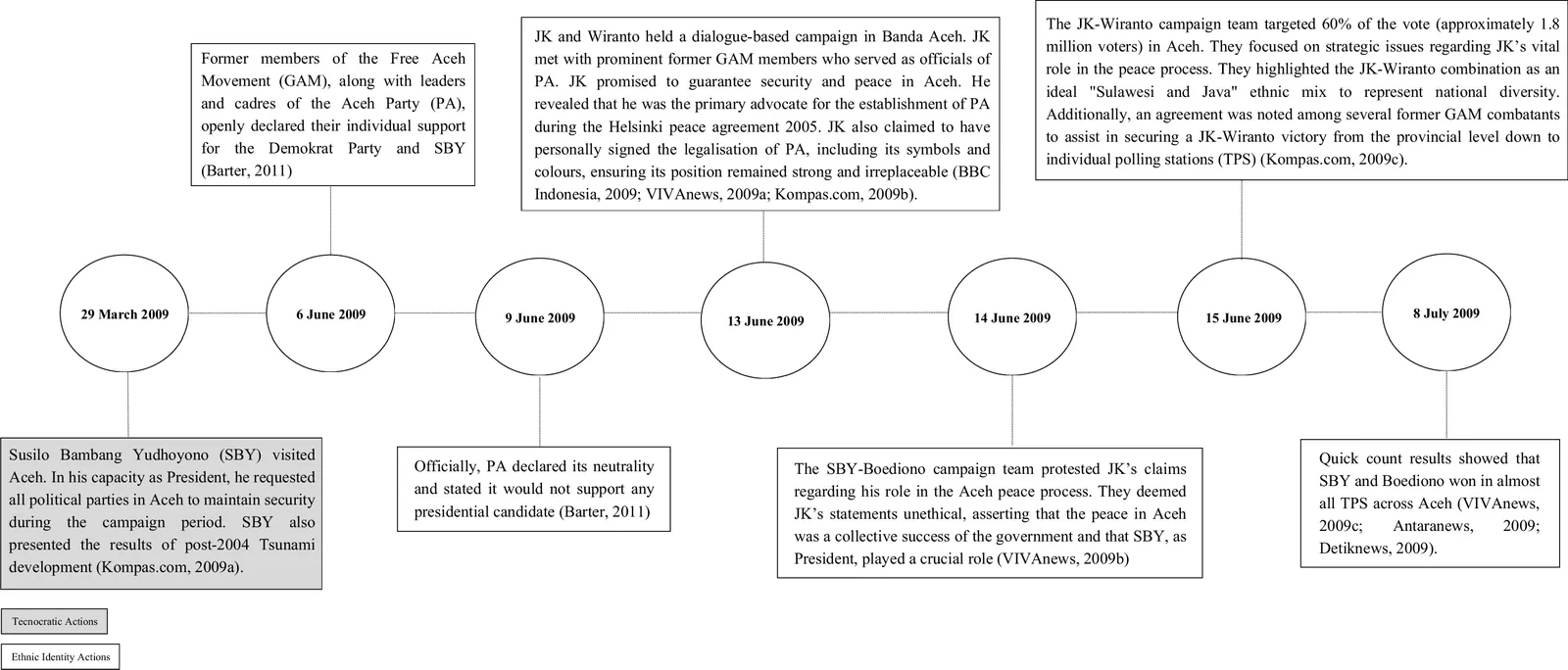

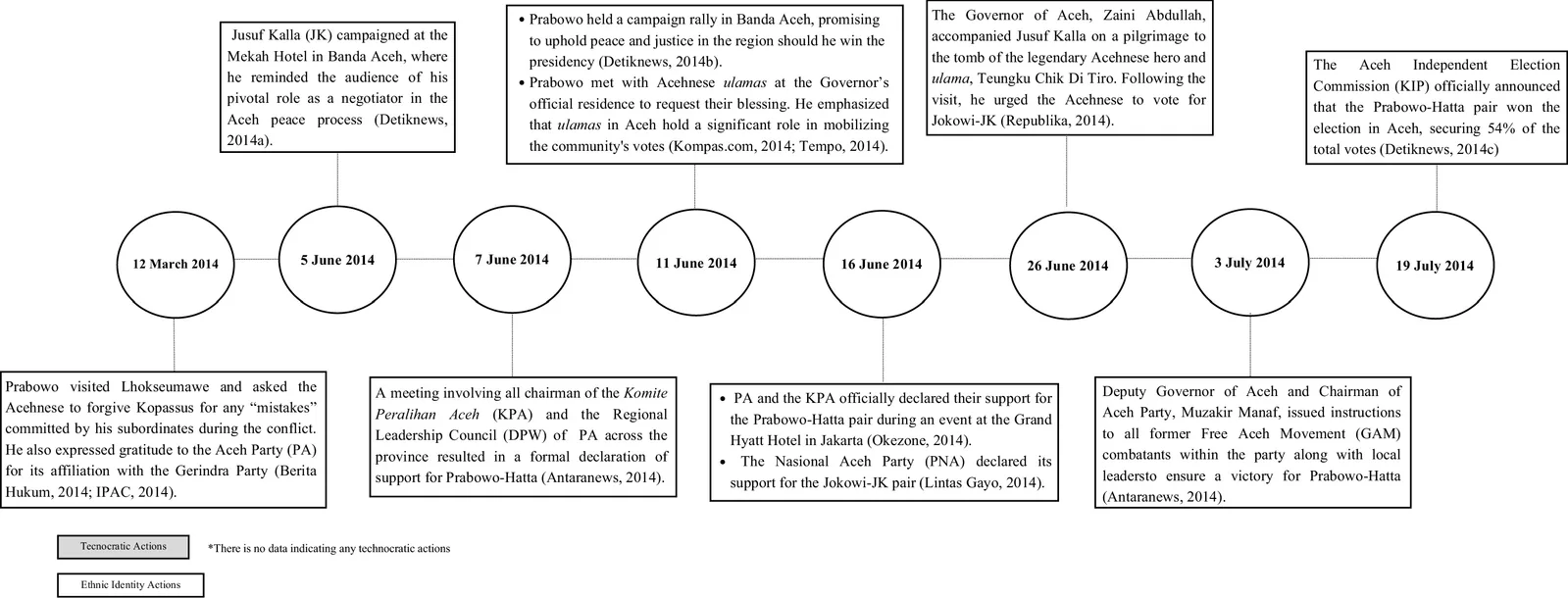

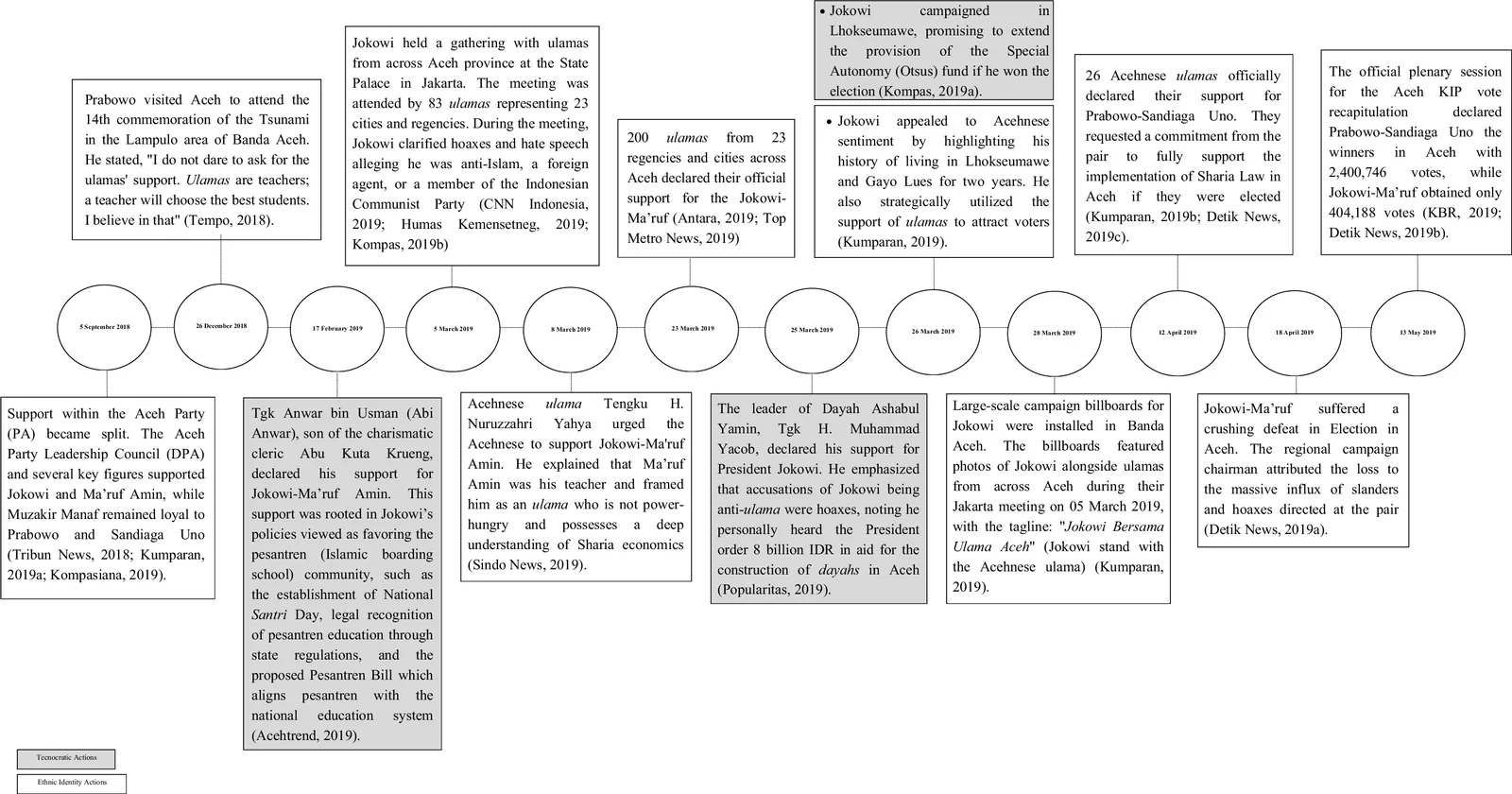

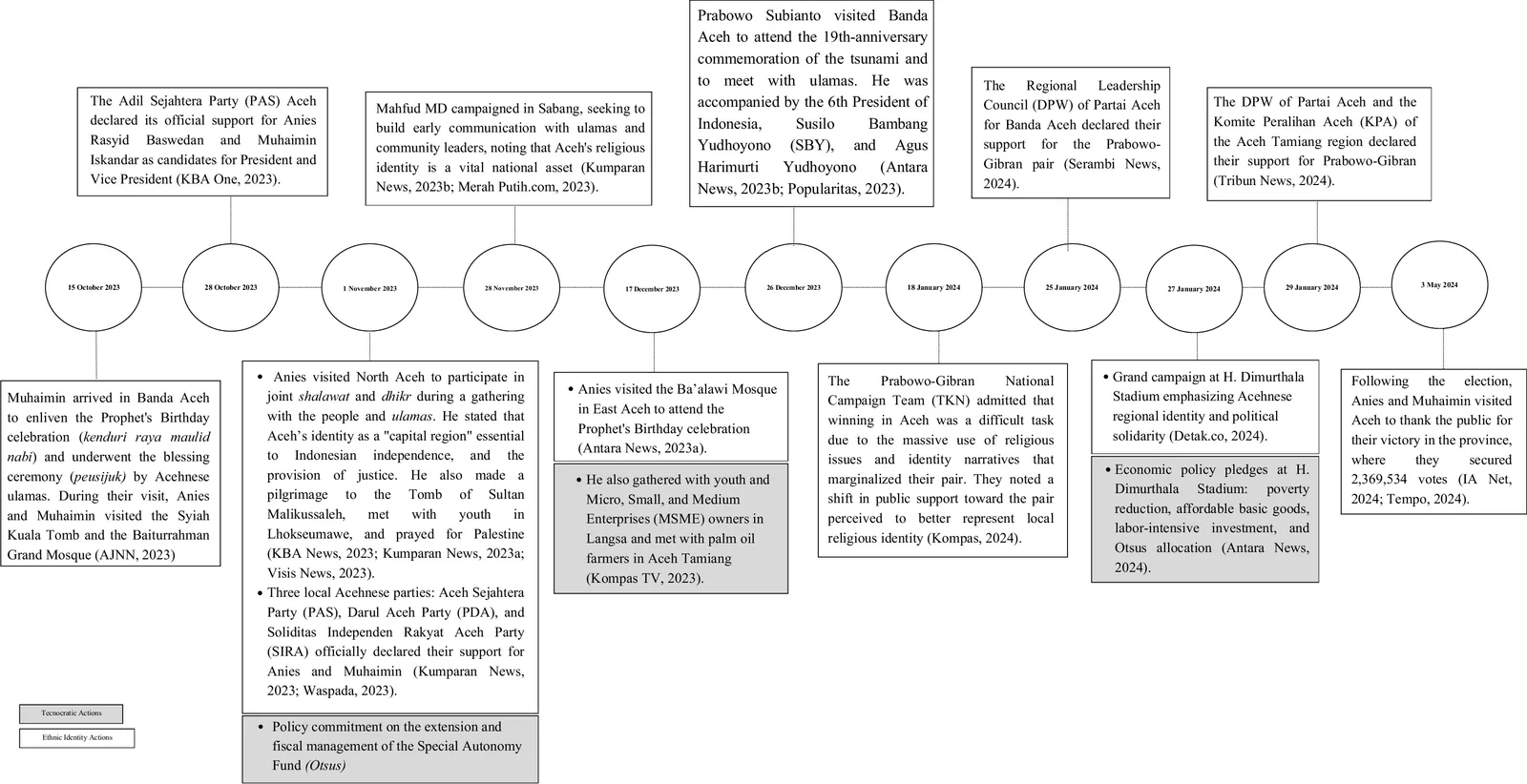
**A**. 2004 Election Cycle. **B**. 2009 Election Cycle. **C**. 2014 Election Cycle. **D**. 2019 Election Cycle. **E**. 2024 Election Cycle.

## Limitations

It is important to clarify that this protocol is explicitly tailored for the case-centric explaining-outcome variant of process tracing [9]. Its primary objective is to provide a contextually grounded, adequate explanation for complex or counterintuitive electoral relationships between cause (X) and outcome (Y) within a specific subnational context during national election cycles. While the seven-step procedural framework can be replicated across other regions, the protocol is not intended to formulate universal theories of voting behavior or to validate a single monolithic theoretical model deductively.

Furthermore, the empirical rigor of this protocol depends heavily on the availability and accessibility of rich secondary data sources (including media archives, campaign speech transcripts, candidate itineraries, and formal political contracts) to reconstruct the intermediate steps of the causal chain. In longitudinal applications, researchers must explicitly account for digitization bias, as digital archives for older election cycles are inherently sparser than those for contemporary ones. Additionally, relying exclusively on secondary documentary sources, without primary elite interviews, poses a clear constraint on reconstructing unrecorded or informal elite bargaining. In settings where archival records are sparse, heavily censored, or unreliable, the systematic identification of diagnostic evidence and subsequent qualitative diagnostic evaluation (Hoop and Smoking Gun Tests) may be hampered.

Finally, successful operationalization demands a high degree of contextual and cultural literacy from the researcher. Categorizing diagnostic evidence, particularly ritualistic and rhetorical evidence, requires an in-depth understanding of regional symbols, socio-religious traditions, and local political vernacular. For instance, a researcher lacking familiarity with regional history might fail to grasp the strategic importance of a candidate's visit to a specific sacred site or participation in traditional legitimization ceremonies (such as *peusijuk*/ blessing rituals in Aceh), thereby misclassifying or overlooking vital diagnostic clues. Consequently, applying this protocol without adequate domain-specific knowledge risks misinterpreting local identity dynamics and compromising the validity of the resulting causal inferences.

## Ethics statements

Ethics approval was not sought for this study because the work did not involve human subjects or data that was not freely available. All ethical guidelines outlined by the journal and the research institution of Universitas Syiah Kuala were adhered to.

## CRediT author statement

**Cut Maya Aprita Sari**: Supervision, Conceptualization, Methodology, and Validation; **Ibnu Phonna Nurdin**: Formal Analysis, Visualization, Writing and Editing; **Annisah Putri**: Investigation, Writing Original Draft, and Data Curation

## Declaration of generative AI and AI-assisted technologies in the manuscript preparation process

During the preparation of this work, the authors used Grammarly and QuillBot to improve the manuscript's readability and language. After using this tool/service, the authors reviewed and edited the content as needed and take full responsibility for the published article.

## Declaration of competing interest

The authors declare that they have no known competing financial interests or personal relationships that could have appeared to influence the work reported in this paper.

## Data Availability

All data generated or analyzed during this study are included in this published article and its supplementary files
