# Supplementary material for: A process-tracing protocol for evaluating ethnic identity mobilization in subnational dynamics of national elections
Source: MethodsX. 2026 Oct 13;17:104203. doi: 10.1016/j.mex.2026.104203 (PMC13694783; doi:10.1016/j.mex.2026.104203)
Supplement: Supplementary file 1 [file mmc1.docx]

**SUPPLEMENTARY TABLE S1. Detailed Candidate List and Source Citations for Election Timelines (2004–2024)**

**S1.1 Candidate Profiles and Key Electoral Schedules Across Five Cycles**

| **Election Cycle** | **Presidential & Vice-Presidential Candidates** | **Nominated Parties / Coalitions** | **Key Electoral Schedules & Campaign Periods** |
| --- | --- | --- | --- |
| 2004 | 1. Wiranto & Salahuddin Wahid | Golkar Party | Campaign Period: June 1 – 1 July 2004  Election Rounds: 5 July & 20 September 2004  Official Results Announcement: 4 October 2004  Inauguration: 20 October 2004 |
|  | 2. Megawati Soekarnoputri & Hasyim Muzadi | PDI-P |  |
|  | 3. Amien Rais & Siswono Yudo Husodo | PAN |  |
|  | 4. Susilo Bambang Yudhoyono & Jusuf Kalla | Democratic Party, PBB, PKPI |  |
|  | 5. Hamzah Haz & Agum Gumelar | PPP |  |
| 2009 | 1. Susilo Bambang Yudhoyono & Boediono | Democratic Party (PD), PKS, PAN, PPP, PKB, PBB | Campaign Period: 2 June – 4 July 2009  Election Day: 8 July 2009  Official Results Announcement: 18 August 2009  Inauguration: 20 October 2009 |
|  | 2. Megawati Soekarnoputri & Prabowo Subianto | PDI-P, Gerindra, PNI Marhaenisme, Labor Party, Sovereignty Party |  |
|  | 3. Jusuf Kalla & Wiranto | Golkar Party, Hanura Party |  |
| 2014 | 1. Prabowo Subianto & Hatta Rajasa | Koalisi Merah Putih (Gerindra, Golkar, PAN, PKS, PPP, PBB, Democratic Party) | Campaign Period: 5 June – 5 July 2014  Election Day: 9 July 2014  Official Results Announcement: 23 July 2014  Inauguration: 20 October 2014 |
|  | 2. Joko Widodo & Jusuf Kalla | Koalisi Indonesia Hebat (PDI Perjuangan, NasDem, PKB, Hanura, PKPI) |  |
| 2019 | 1. Joko Widodo & Ma'ruf Amin | PDI Perjuangan, Golkar, PKB, NasDem, PPP, Hanura, PKPI | Campaign Period: 23 September 2018 – 13 April 2019  Election Day: 17 April 2019  Official Results  Announcement: 21 May 2019  Inauguration: 20 October 2019 |
|  | 2. Prabowo Subianto & Sandiaga Uno | Gerindra, Democratic Party, PAN, PKS |  |
| 2024 | 1. Anies Baswedan & Muhaimin Iskandar | NasDem Party, PKB, PKS, Ummat Party | Campaign Period: 28 November 2023 – 10 February 2024  Election Day: 14 February 2024  Official Results Announcement: March 20, 2024  Inauguration: 20 October 2024 |
|  | 2. Prabowo Subianto & Gibran Rakabuming Raka | Gerindra, Golkar, Democratic Party, PAN, PSI, PBB, Garuda Party |  |
|  | 3. Ganjar Pranowo & Mahfud MD | PDI Perjuangan, PPP, PERINDO, Hanura Party |  |

**S1.2 Source Citations for Election Timelines (2004–2024)**

| **Election Cycle** | **Author/Source** | **URL** |
| --- | --- | --- |
| **2004** | (Nurhasim, 2004) | <https://doi.org/10.58823/jham.v10i10.82> |
|  | (Detik News, 2004a) | <https://news.detik.com/berita/d-160409/hasyim-muzadi-kandidat-pilpres-pertama-kampanye-di-aceh> |
|  | (Liputan6, 2004c) | <https://www.liputan6.com/news/read/80065/amien-rais-berkampanye-di-banda-aceh> |
|  | (Liputan6, 2004a) | <https://www.liputan6.com/news/read/81422/catatan-kecil-kampanye-sby-jk> |
|  | (Tempo, 2004) | <https://www.tempo.co/arsip/amien-makan-siang-di-pasar-aceh-2009060> |
|  | (Gade, 2006) | <https://eradzie.wordpress.com/2006/10/29/ketika-ulama-aceh-minta-jatah-menteri-agama/> |
|  | (Abdullah & Salim, 2014) | <https://doi.org/10.58823/jham.v10i10.82> |
|  | (Abdullah & Salim, 2014) | <https://doi.org/10.58823/jham.v10i10.82> |
|  | (Liputan6, 2004b) | <https://www.liputan6.com/news/read/84427/megawati-di-aceh-disambut-puteh> |
|  | (Detiknews, 2004b) | <https://news.detik.com/berita/d-194111/mega-hanya-aceh-yang-akui-presiden-perempuan> |
|  | (Aspinall, 2005) | <https://doi.org/10.5367/0000000054604515> |
|  | (Liputan6, 2004d) | <https://doi.org/10.14203/jpp.v1i1.376> |
|  | (Detik News, 2004c) | <https://news.detik.com/berita/d-211097/sby-dulang-80-suara-di-aceh> |
| **2009** | (Kompas.com, 2009a) | <https://news.kompas.com/read/2009/03/29/10575216/sby-minta-parpol-di-aceh-jaga-keamanan> |
|  | (Barter, 2011) | <https://doi.org/10.5728/indonesia.91.0113> |
|  | (Serambi Indonesia, 2009) | <https://doi.org/10.5728/indonesia.91.0113> |
|  | (BBC Indonesia, 2009) | <https://www.bbc.co.uk/indonesian/news/story/2009/06/printable/090613_capres.shtml> |
|  | (VIVAnews, 2009a) | <https://www.viva.co.id/arsip/65878-sore-ini-jk-temui-tokoh-dan-partai-aceh> |
|  | (Kompas.com, 2009b) | <https://lifestyle.kompas.com/read/2009/06/13/10062068/jk-janji-penuhi-100-persen-perdamaian-aceh> |
|  | (VIVAnews, 2009b) | <https://www.viva.co.id/arsip/66424-tim-sukses-sby-protes-klaim-jk-soal-aceh> |
|  | (Kompas.com, 2009c) | <https://regional.kompas.com/read/2009/06/15/20334324/pasangan-jk-win-targetkan-60-persen-perolehan-suara> |
|  | (Antaranews, 2009) | <https://www.antaranews.com/berita/146757/hasil-hitung-cepat-menangkan-sby-boediono-satu-putaran> |
|  | (Detiknews, 2009) | https://news.detik.com/pemilu/d-1161883/peran-jk-hanya-diketahui-politisi-sby-menang-90-di-aceh |
|  | (VIVAnews, 2009c) | <https://www.viva.co.id/arsip/73485-sby-unggul-di-aceh-karena-kedekatan> |
| **2014** | (Berita Hukum, 2014) | <https://m.beritahukum.com/detail_berita.php?judul=Prabowo+Minta+Maaf+Kesalahan+Kopassus+Semasa+Konflik+di+Aceh&subjudul=Prabowo+Subianto> |
|  | (IPAC, 2014) | <https://understandingconflict.sgp1.digitaloceanspaces.com/dashboard/5c13969ed166423835ae4291ba544073.pdf> |
|  | (Tribunnews, 2014) | <https://www.tribunnews.com/pemilu-2014/2014/06/06/jokowi-jk-yakin-dukungan-mayoritas-rakyat-aceh> |
|  | (Detiknews, 2014a) | <https://www.detik.com/pemilu/kampanye-di-aceh-surya-paloh-puji-jasa-jk-atasi-konflik-gam> |
|  | (Antaranews, 2014) | <https://www.antaranews.com/berita/442282/partai-aceh-tegaskan-dukung-prabowo-hatta> |
|  | (Detiknews, 2014b) | <https://news.detik.com/berita/d-2605657/prabowo-bicara-masa-damai-dan-keadilan-di-aceh> |
|  | (Tempo, 2014) | <https://www.tempo.co/arsip/hari-ini-prabowo-kampanye-di-aceh-1521891> |
|  | (Kompas.com, 2014) | <https://nasional.kompas.com/read/2014/06/11/1937306/Prabowo.Minta.Dukungan.Ulama.Aceh.> |
|  | (Tempo, 2014) | <https://www.tempo.co/arsip/hari-ini-prabowo-kampanye-di-aceh-1521891> |
|  | (Okezone, 2014) | <https://news.okezone.com/read/2014/06/16/568/999659/partai-aceh-resmi-dukung-prabowo-hatta> |
|  | (Lintas Gayo, 2014) | <https://lintasgayo.com/49206/partai-aceh-ke-prabowo-pna-ke-joko-widodo-pda-ke-mana/> |
|  | (Republika, 2014) | <https://republika.co.id/berita/pemilu/menuju-ri-1/14/06/26/n7rh5d-ini-alasan-jk-dua-kali-kampanye-di-aceh> |
|  | (Antaranews, 2014) | <https://www.antaranews.com/berita/442282/partai-aceh-tegaskan-dukung-prabowo-hatta> |
|  | (Detiknews, 2014c) | <https://news.detik.com/berita/d-2641810/raih-54-suara-prabowo-hatta-menang-di-aceh> |
|  | (IPAC, 2014) | <https://understandingconflict.sgp1.digitaloceanspaces.com/dashboard/5c13969ed166423835ae4291ba544073.pdf> |
| **2019** | (Tribun News, 2018) | <https://www.tribunnews.com/pilpres-2019/2018/09/03/dua-elite-partai-aceh-beda-pilihan-muzakir-manaf-setia-pada-prabowo-abu-razak-merapat-ke-jokowi> |
|  | (Kompasiana, 2019) | <https://www.kompasiana.com/amirmahmud8043/5b8f9f35ab12ae7ac775f9f4/internal-partai-aceh-pa-tidak-sepaham-dalam-pilpres-2019> |
|  | (Kumparan, 2019a) | [https://kumparan.com/acehkini/adu-kuat-jokowi-dan-prabowo-di-aceh-1qmMIIu0XEW/full ;](https://kumparan.com/acehkini/adu-kuat-jokowi-dan-prabowo-di-aceh-1qmMIIu0XEW/full%20;) |
|  | (Tempo, 2018) | <https://www.tempo.co/politik/kunjungi-ponpes-di-aceh-prabowo-tak-berani-minta-dukungan-ulama-785373> |
|  | (Aceh Trend, 2019) | <https://www.acehtrend.com/news/putra-abu-kuta-krueng-dukung-jokowi-dua-periode/index.html> |
|  | (CNN Indonesia, 2019) | <https://www.cnnindonesia.com/nasional/20190305161311-32-374696/ulama-aceh-berharap-jokowi-kembali-memimpin-indonesia> |
|  | (Humas Kemensetneg, 2019) | <https://www.setneg.go.id/baca/index/bertemu_presiden_ulama_aceh_sepakat_dukung_pemilu_damai> |
|  | (Kompas, 2019b) | <https://nasional.kompas.com/read/2019/03/05/21121901/ulama-ulama-aceh-akan-bantu-jokowi-klarifikasi-sejumlah-isu-miring> |
|  | (SindoNews, 2019) | <https://nasional.sindonews.com/berita/1385246/12/ulama-aceh-ajak-pilih-jokowi-maruf-pada-pilpres-ini-alasannya> |
|  | (Antara, 2019) | <https://www.antaranews.com/berita/814549/ulama-aceh-deklarasi-dukung-jokowi-maruf-amin> |
|  | (Top Metro News, 2019) | <https://topmetro.news/59616/deklarasi-dukungan-ulama-aceh/> |
|  | (Popularitas, 2019) | <https://popularitas.com/berita/pimpinan-dayah-ashabul-yamin-semua-sikap-positif-ada-di-jokowi/> |
|  | (Kompas, 2019a) | <https://www.kompas.id/artikel/jokowi-janji-perpanjang-dana-otsus-aceh> |
|  | (Kumparan, 2019a) | <https://kumparan.com/acehkini/adu-kuat-jokowi-dan-prabowo-di-aceh-1qmMIIu0XEW/full> |
|  | (Kumparan, 2019a) | <https://kumparan.com/acehkini/adu-kuat-jokowi-dan-prabowo-di-aceh-1qmMIIu0XEW/full> |
|  | (Kumparan, 2019b) | <https://kumparan.com/kumparannews/ulama-aceh-teken-mou-dukungan-untuk-prabowo-sandi-1qs9rOy6llM/3> |
|  | (Detik News, 2019c) | <https://news.detik.com/berita/d-4507996/26-ulama-aceh-teken-nota-kesepahaman-dengan-prabowo-sandi> |
|  | (Detik News, 2019a) | <https://news.detik.com/berita/d-4516094/jokowi-kalah-telak-di-aceh-tkd-kami-tak-mampu-bendung-hoax> |
|  | (KBR, 2019) | [https://kbr.id/articles/indeks/pleno_kip_aceh__prabowo_sandi_menang_telak](https://kbr.id/articles/indeks/pleno_kip_aceh__prabowo_sandi_menang_telak%20) |
|  | (Detik News, 2019b) | <https://news.detik.com/berita/d-4529072/rekap-suara-di-banda-aceh-rampung-prabowo-sandiaga-menang-telak> |
| **2024** | (AJNN, 2023) | <https://www.ajnn.net/news/anies-baswedan-dan-cak-imin-akan-datang-ke-aceh/index.html> |
|  | (KBA One, 2023) | <https://www.kba.one/news/partainya-para-ulama-di-aceh-deklarasi-pilih-pasangan-anies-cak-imin/index.html> |
|  | (KBA News, 2023) | <https://kbanews.com/hot-news/kunjungan-ke-tanah-pasee-50-ribu-warga-aceh-siap-sambut-anies-baswedan-besok/> |
|  | (Visi News, 2023) | <https://visi.news/di-aceh-anies-berziarah-ke-makam-pendiri-kerajaan-samudera-pasai/> |
|  | (Waspada, 2023) | <https://waspadaaceh.com/3-parlok-aceh-deklarasi-dukung-anies-gus-imin-di-pilpres-2024/> |
|  | (Kumparan News, 2023b) | <https://kumparan.com/kumparannews/masyarakat-aceh-miliki-kecenderungan-memilih-anies-21Vq5VieWn0/full> |
|  | (Kumparan News, 2023a) | <https://kumparan.com/kumparannews/mahfud-ungkap-alasan-pilih-aceh-jadi-lokasi-pertama-kampanye-21fF9mrztQT/full> |
|  | (Merah Putih.com, 2023) | <https://www.merahputih.com/post/read/hari-pertama-kampanye-mahfud-kunjungi-sabang-aceh-dan-ganjar-ke-merauke> |
|  | (Antara News, 2023a) | <https://aceh.antaranews.com/berita/350376/sambut-capres-anies-baswedan-ribuan-masyarakat-aceh-timur-padati-masjid> |
|  | (Kompas TV, 2023) | <https://www.kompas.tv/video/469792/sambangi-provinsi-aceh-anies-kunjungi-masjid-ba-alawi> |
|  | (Antara News, 2023b) | <https://kalsel.antaranews.com/berita/399645/prabowo-ke-aceh-pada-hari-ke-28-kampanye> |
|  | (Popularitas, 2023) | <https://popularitas.com/berita/kampenye-di-aceh-prabowo-janjikan-bangun-poltek-unggulan-jika-jadi-presiden/> |
|  | (Kompas, 2024) | <https://nasional.kompas.com/read/2024/01/18/22144981/akui-berat-menangkan-prabowo-gibran-di-aceh-tkn-ada-isu-agama-yang-dibawa> |
|  | (Serambi News, 2024) | <https://aceh.tribunnews.com/2024/01/25/pa-banda-aceh-deklarasi-dukung-prabowo-gibran> |
|  | (Antara News, 2023c) | <https://aceh.antaranews.com/berita/352911/kampanye-akbar-anies-serukan-semangat-perubahan-di-hadapan-rakyat-aceh> |
|  | (Detak.co, 2024) | <https://detak.co/kategori/nasional/kampanye-akbar-di-stadion-h-dimurthala-anies-aceh-tempat-lahirnya-pejuang-perubahan> |
|  | (Tribun News, 2024) | <https://aceh.tribunnews.com/2024/01/29/pa-dan-kpa-aceh-tamiang-deklarasi-dukungan-prabowo-gibran> |
|  | (IA Net, 2024) | <https://www.infoaceh.net/2024/05/03/anies-cak-imin-kunjungi-aceh-usai-pilpres-ucapkan-terima-kasih-ke-warga/> |
|  | (Tempo, 2024) | <https://www.tempo.co/politik/menang-telak-di-aceh-saat-pilpres-2024-anies-terima-kasih-orang-orang-pemberani-62313> |
